# Repellent Plants Provide Affordable Natural Screening to Prevent Mosquito House Entry in Tropical Rural Settings—Results from a Pilot Efficacy Study

**DOI:** 10.1371/journal.pone.0025927

**Published:** 2011-10-12

**Authors:** Frank C. Mng'ong'o, Joseph J. Sambali, Eustachkius Sabas, Justine Rubanga, Jaka Magoma, Alex J. Ntamatungiro, Elizabeth L. Turner, Daniel Nyogea, Jeroen H. J. Ensink, Sarah J. Moore

**Affiliations:** 1 Concern Worldwide Tanzania, Dar es Salaam, Tanzania; 2 Disease Control Department, London School of Hygiene and Tropical Medicine, London, United Kingdom; 3 Biomedical and Environmental Group, Ifakara Health Institute, Ifakara, Tanzania; 4 Department of Geography, Durham University, Durham, United Kingdom; 5 Department of Medical Statistics, London School of Hygiene and Tropical Medicine, London, United Kingdom; Johns Hopkins University, United States of America

## Abstract

Sustained malaria control is underway using a combination of vector control, prompt diagnosis and treatment of malaria cases. Progress is excellent, but for long-term control, low-cost, sustainable tools that supplement existing control programs are needed. Conventional vector control tools such as indoor residual spraying and house screening are highly effective, but difficult to deliver in rural areas. Therefore, an additional means of reducing mosquito house entry was evaluated: the screening of mosquito house entry points by planting the tall and densely foliated repellent plant *Lantana camara* L. around houses. A pilot efficacy study was performed in Kagera Region, Tanzania in an area of high seasonal malaria transmission, where consenting families within the study village planted *L. camara* (Lantana) around their homes and were responsible for maintaining the plants. Questionnaire data on house design, socioeconomic status, malaria prevention knowledge, attitude and practices was collected from 231 houses with Lantana planted around them 90 houses without repellent plants. Mosquitoes were collected using CDC Light Traps between September 2008 and July 2009. Data were analysed with generalised negative binomial regression, controlling for the effect of sampling period. Indoor catches of mosquitoes in houses with Lantana were compared using the Incidence Rate Ratio (IRR) relative to houses without plants in an adjusted analysis. There were 56% fewer *Anopheles gambiae* s.s. (IRR 0.44, 95% CI 0.28–0.68, p<0.0001); 83% fewer *Anopheles funestus* s.s. (IRR 0.17, 95% CI 0.09–0.32, p<0.0001), and 50% fewer mosquitoes of any kind (IRR 0.50, 95% CI 0.38–0.67, p<0.0001) in houses with Lantana relative to controls. House screening using Lantana reduced indoor densities of malaria vectors and nuisance mosquitoes with broad community acceptance. Providing sufficient plants for one home costs US $1.50 including maintenance and labour costs, (30 cents per person). *L. camara* mode of action and suitability for mosquito control is discussed.

## Introduction

In order to achieve the sustained malaria control needed for elimination, new tools are required to help maintain and improve the effectiveness of currently available tools (Long lasting insecticidal nets (LLINs) and indoor residual spraying (IRS)) and new vector-targeted tools are needed that can be used to interrupt transmission in situations where those existing tools cannot reach [1].

Current vector control with LLINS and IRS has dramatically reduced the malaria burden and under-five mortality in participating countries throughout Africa [2], including Tanzania [3]. LLINs are the most cost effective means of malaria prevention in highly disease endemic areas [4]. However, they still cost $5 each [5] plus additional marketing and delivery cost, while they have a finite lifespan. In order to be effective, LLINs require a high degree of (nightly) user compliance, which is not always achieved [6]. IRS does not require user compliance but it is costly and logistically challenging to undertake in remote rural areas, and needs to be undertaken indefinitely at high coverage rates in order to be effective in highly endemic settings [7]. Cheaper technologies that supplement these tools would be of benefit for introduction into Kagera region in Tanzania where the study took place. Kagera is one of the most remote regions in Tanzania, and it is extremely poor with 33% of households below the basic needs poverty line [8]. It is also the most highly malaria endemic region of Tanzania [9], thus posing a great challenge for long-term malaria control.

The effectiveness of any vector control intervention depends on four factors: 1) unit efficacy i.e. the number of disease events prevented per unit resource outlay, 2) the duration of that protection, 3) community acceptance and uptake, 4) individual, community or programmatic adherence. Therefore, ideal vector control technologies protect large numbers of people for a long time at a realistically affordable price. Additionally, a practical vector control intervention should be simple and unobtrusive, requiring little investment or effort from the end-users.

An example of a technology that 1) protects multiple individuals for prolonged periods of time, 2) is widely accepted and 3) has no need for regular user compliance, is house screening [10]. There is strong data showing that house modification through physical screening [11] and insecticide treated curtains [12], [13], [14], [15] lowers malaria transmission among users in Africa. Whilst their uptake is high in urban areas [16], the cost of $25 per household for those living in poorer rural areas may be prohibitive, and it may not be possible to fix screens onto traditional mud brick houses. The focus of this research was to investigate an alternative means of household protection from mosquitoes using repellent plants.

We selected a fast growing, densely foliated plant that is used locally to repel mosquitoes to test its potential as a physical and chemical barrier to prevent mosquito house entry. This approach was used because plants are extremely cheap to produce and are self-sustaining. Therefore, after an initial outlay to produce the plants in a nursery, a house could be protected indefinitely with the only adherence required by householders being the maintenance of the plants. We identified plants that were used locally to repel mosquitoes as these are already accepted within the community where the study took place. *Lantana camara* L. (Lantana) was selected as the plant to be tested in a pilot community study to evaluate its potential to reduce mosquito house entry. The paper reports the excellent efficacy of Lantana in reducing mosquito house entry, together with a broader discussion of its interesting modes of action and suitability for mosquito control.

## Results

### Plants used locally to repel mosquitoes

**Figure 1:**
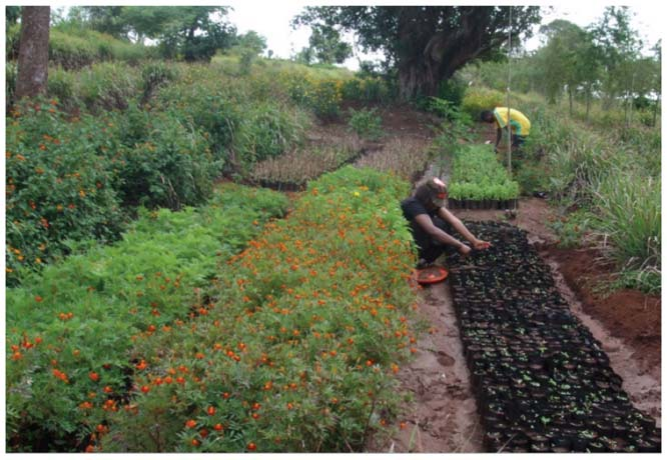
Concern Plant Nursery in Ngara.

**Figure 2:**
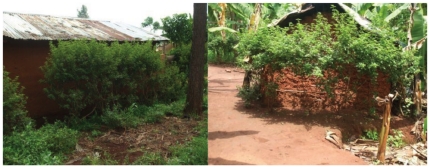
Lantana planted around a house in the study village. The plants grew rapidly to cover the sides of the houses where mosquitoes enter through small openings and eaves.

Several plants were used locally to repel mosquitoes (Table S1): wild sage, Lantana (*L. camara* L.); Mwarobaini, neem (*Azadirachta indica* A. Juss); mchaichai, Lemongrass (*Cymbopogon citratus* L.); nabhengele – several members of the genus *Ocimum* were used including *Ocimum americanum* L., *Ocimum kilimandscharicum* Guerke and *Ocimum suave* Willd. These plants had been established in a nursery by Concern Worldwide Tanzania (Figure 1). Of the plants Lantana was selected for the pilot study based upon its size and vigour (Figure 2), year round growth and known repellency towards the African malaria vector *Anopheles gambiae* s.s.

### Baseline characteristics of households with and without Lantana

The baseline survey was conducted in September 2008. At that time, many people taking part in the evaluation had lived in the study village for fewer than 5 years (45%). This reflects the changing population in Ngara district due to an influx of refugees from Burundi, many of which have now settled in Tanzania and internal migration of Tanzanians seeking land. The family unit was similar to Tanzanian averages [17] with 1–2 small children under five, 2–3 children between 5 and 18 years and 2 adults per household. Most respondents (60%) were aged between 18 and 35 years, and the majority (70%) were female. Most participants had low levels of education: 34% had never attended school and 60% had attended primary school only. Most people were subsistence farmers (72%) and the remainder worked as labourers, or ran small businesses such as tailors or mat makers. Few participants owned assets such as mobile phones (30%), television set (3%) and a bicycle (36%) indicating low disposable income. The exception was a radio, owned by 66% of people. These factors did not vary between households with and without Lantana.

### Factors that may influence mosquito house-entry

Factors in house design and occupants' behaviour that may influence mosquito house entry were analysed to see whether there was an underlying difference between those houses with and without Lantana that would bias or mask the effect of the plants (Table 1). There was little difference in house-occupancy, number of bednets owned or bednet use. However, there were some important differences in house design: a greater proportion of houses in the Lantana group had thatch roofs that provide refuge for blood-fed mosquitoes (62% vs. 42%), and a smaller proportion of houses in the Lantana group had open eaves that are favoured as house entry points by host-seeking malaria mosquitoes (28% vs. 46%). The non-Lantana group had a greater proportion of houses that had extensive smoke stains that may reduce indoor mosquito density (42% vs. 27%) and more people in the non-plant group kept livestock indoors that may increase indoor mosquito density (57% vs. 38%). As the two groups showed variation in factors that are important predictors of indoor mosquito density, mosquito count data were subjected to univariable analysis (Table 2), followed by multivariable analysis of those factors that did affect indoor mosquito counts (Table 3).

**Table 1:** Number and percentage of houses in the study with and without Lantana that have factors assumed to influence indoor mosquito density.

Risk Factor		No plants around houseN = 90 (%)	Lantana around houseN = 231(%)	χ 2 testP value
Number of people living in the house	1–4	40 (44.4)	104 (45)	N.S.
	5–8	43(47.8)	112 (48.5)	
	9–12	7 (7.8)	15 (6.5)	
Number of bednets owned	0	56 (62)	123 (53.3)	N. S.
	1–3	33 (36.7)	107 (46.3)	
	4–5	1 (1)	1 (0.4)	
Number of people using bednets the previous night	0	60 (66.7)	130 (56.3)	N.S.
	1–3	20 (22.2)	72 (31.2)	
	4–6	9 (10)	25 (10.8)	
	7–11	1 (1)	4 (1.7)	
Place of cooking	Indoors	72 (80)	172 (74.5)	N.S.
	Outdoors	18 (20)	59 (25.5)	
Keep a fire burning overnight	No	84 (94.4)	218 (95.2)	N.S.
	Yes	5 (5.6)	11 (4.8)	
Roof material	Thatch	38 (42.2)	140 (62)	P = 0.001
	Tin	52 (57.8)	86 (38)	
Eave gap	Closed	49 (54.4)	166 (71.9)	P = 0.003
	Open	41 (45.6)	65 (28.1)	
Smoke stains in the house	None	32 (35.6)	126 (54.5)	P = 0.007
	Some <⅓	20 (22.2)	42 (18.2)	
	Extensive >⅓	38 (42.2)	63 (27.3)	
Where livestock stay at night	No livestock	27 (30)	94 (40.7)	P = 0.01
	Indoors	51 (56.7)	88 (38.1)	
	Outdoors	12 (13.3)	49 (21.2)	

**Table 2:** Association between mosquito counts and factors assumed to influence indoor mosquito density as measured in incidence rate ratios (IRR) from univariable negative binomial regression models.

				*Anopheles gambiae* s.l.			*Anopheles funestus* s.s.			All mosquitoes		
Factor	Type of variable	N	Reference	IRR	95% CI	p	IRR	95% CI	p	IRR	95% CI	p
Plant Used	Factor	321	No plants	1		0.002	1		<0.0001	1		<0.0001
			Lantana	0.48	0.30–0.76		0.17	0.09–0.30		0.54	0.40–0.73	
People in H/Hold	Continuous	321	per 1 person	1.03	0.94–1.13	N.S.	1.02	0.91–1.16	N.S.	1.007	0.94–1.08	N.S.
No slept under nets last night	Continuous	321	per 1 net	0.93	0.83–1.04	N.S.	1.22	1.03–1.44	0.02	1.02	0.94–1.12	N.S.
Bednets owned	Continuous	321	per 1 net	0.59	0.38–0.93	0.02	1.87	0.98–3.58	N.S.	0.91	0.67–1.22	N.S.
Roof material	Factor	316	Tin	1		N.S.	1		N.S.	1		N.S.
			Thatch	0.95	0.60–1.52		0.83	0.43–1.59		0.99	0.74–1.34	
Eaves	Factor	321	Closed	1		N.S.	1		N.S.	1		N.S.
			Open	1.36	0.84–2.20		1.35	0.65–2.81		1.28	0.93–1.78	
Place of cooking	Factor	321	Indoors	1		N.S.	1		N.S.	1		N.S.
			Outdoors	1.27	0.73–2.21		0.74	0.38–1.43		1.17	0.82–1.67	
Burn a fire overnight	Factor	318	No fire	1		N.S.	1		N.S.	1		N.S.
			Fire	1.52	0.50–4.63		1.04	0.42–2.56		1.06	0.60–1.89	
VisibleSmoke Stains	Factor	321	None	1			1			1		
			Some	0.78	0.43–1.41	N.S.	0.85	0.36–2.04	N.S.	0.89	0.60–1.31	N.S.
			Extensive	0.79	0.46–1.33	N.S.	0.80	0.38–1.70	N.S.	0.73	0.53–0.99	0.05
Livestock overnight in house	Factor	321	None	1			1			1		
			Indoors	1.80	1.05–3.09	0.03	1.10	0.47–2.57	N.S.	1.26	0.89–1.78	N.S.
			Outdoors	1.05	0.58–1.91	N.S.	0.60	0.23–1.53	N.S.	1.02	0.71–1.48	N.S.
Sampling period	Factor	321	Sept–Dec '08	1			1			1		
			Jan–April '09	0.39	0.24–0.64	<0.0001	2.99	1.00–8.93	0.049	1.08	0.78–1.49	N.S.
			May–July '09	0.27	0.14–0.53	<0.0001	7.04	2.29–21.69	0.001	1.28	0.82–2.01	N.S.

**Table 3:** Association between mosquito counts and factors assumed to influence indoor mosquito density as measured in incidence rate ratios (IRR) from multivariable negative binomial regression models.

Mosquito	Risk Factor	IRR	Std. Err.	z	P	95% C.I.	% Efficacy
*Anopheles gambiae* s.s.N = 321	No plants	1					56%
	Lantana	0.44	0.099	−3.62	<0.0001	0.278–0.683	
	Bednets in h/hold	0.762	0.092	−2.25	0.024	0.602–0.965	
	Sept–Dec '08	1					
	Jan–April '09	0.39	0.094	−3.91	<0.0001	0.242–0.624	
	May–July '09	0.26	0.096	−3.64	<0.0001	0.128–0.539	
*Anopheles funestus* s.s.N = 321	No plants	1					83%
	Lantana	0.174	0.055	−5.50	<0.0001	0. 094–0.323	
	Sept–Dec '08	1					
	Jan–April '09	3.619	2.092	2.22	0.026	1.165–11.239	
	May–July '09	6.017	3.534	3.06	0.002	1.903–19.025	
Total mosquitoesN = 318	No plants	1					50%
	Lantana	0.503	0.072	−4.77	<0.0001	0.380–0.667	
	No smoke stains	1					
	Some	0.763	0.131	−1.58	N.S.	0.546–1.067	
	Extensive	0.648	0.104	−2.70	0.007	0.474–0.888	

### Effect of Lantana camara on indoor mosquito density

Between September 2008 and July 2009, 1529 mosquitoes were collected in 321 households with Center for Disease Control Light Trap (CDC LT). Of these, 367 were identified morphologically to be *An. gambiae* s.l. of which 305 specimens were tested by PCR for *An. gambiae* s.s., *An. arabiensis* and *An. quadriannulatus*. Successful amplification was low due to poor handling of specimens at the beginning of the study, however of those that amplified 79% were *An. gambiae* s.s. (26/33 successful amplifications) and 21% were *An. arabiensis* (7/33 successful amplifications), no mosquitoes amplified for *An. quadriannulatus*. Also collected were 270 *An. funestus* sl. of which 99% were *An. funestus* s.s. (258/260 successful amplifications) and 1% *An. parensis* (2/260 successful amplifications); 52 other *Anopheles* including *An. coustani*, *An. pharoensis* and *An. tenebrosus*, along with 1529 non-anophelines including *Culex quinquefasciatus*, *Coquillettidia aurites*, *Coquillettidia versicolor* and small numbers of unidentified *Culex* (*Culex*) mosquitoes.

**Figure 3:**
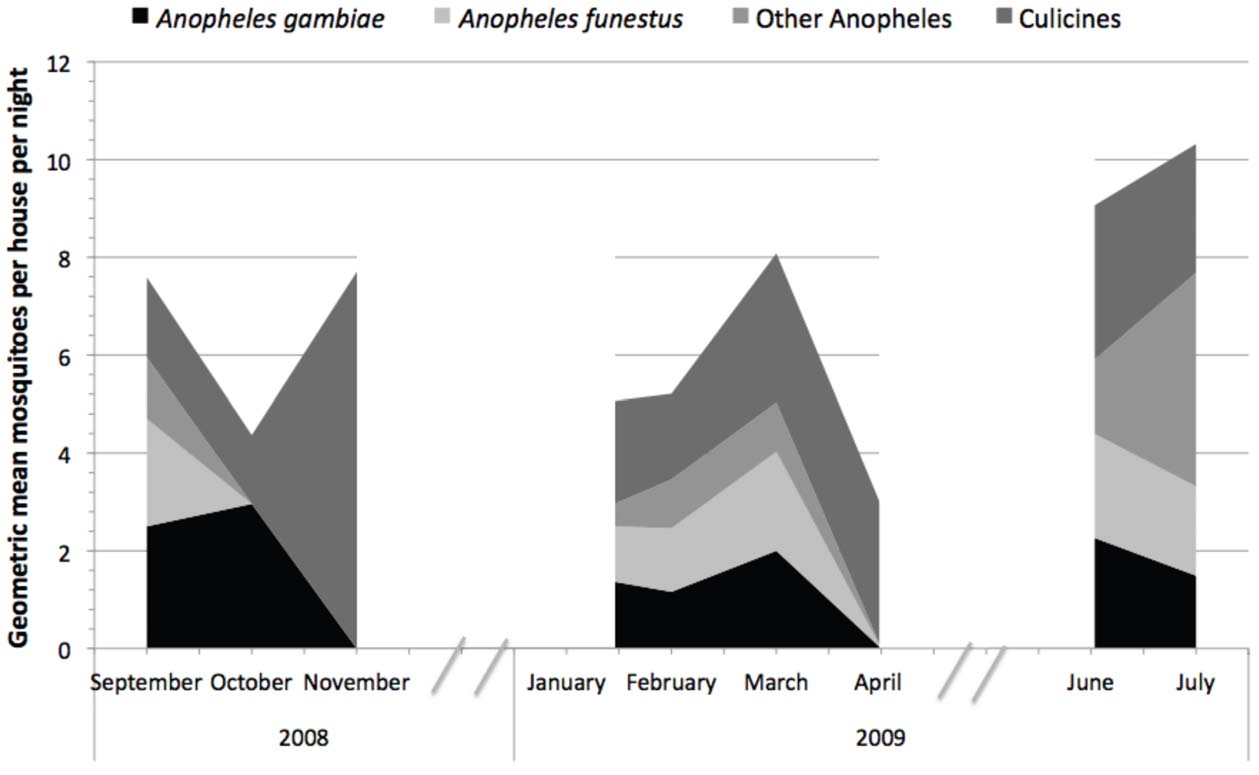
Geometric mean mosquitoes collected per night using CDC LT during the three sampling periods 1) September–November 2008; 2) January to April 2009; 3) June–July 2009. Areas are stacked to show how the relative composition of mosquitoes changes through time.

Analyses were controlled for sampling period as there was a difference in the relative proportion of vector and non-vector mosquitoes (Figure 3) that is clearly dependent on rainfall affecting the abundance of suitable breeding sites. In univariable analysis there was a protective effect of having Lantana planted around homes for *An. gambiae* s.s., *An. funestus* s.s. and total mosquitoes (Table 2). There was no significant effect on indoor densities of culicine mosquitoes in houses with Lantana (IRR 0.95, 95% C.I. 0.67–1.37 p = 0.81).

**Figure 4:**
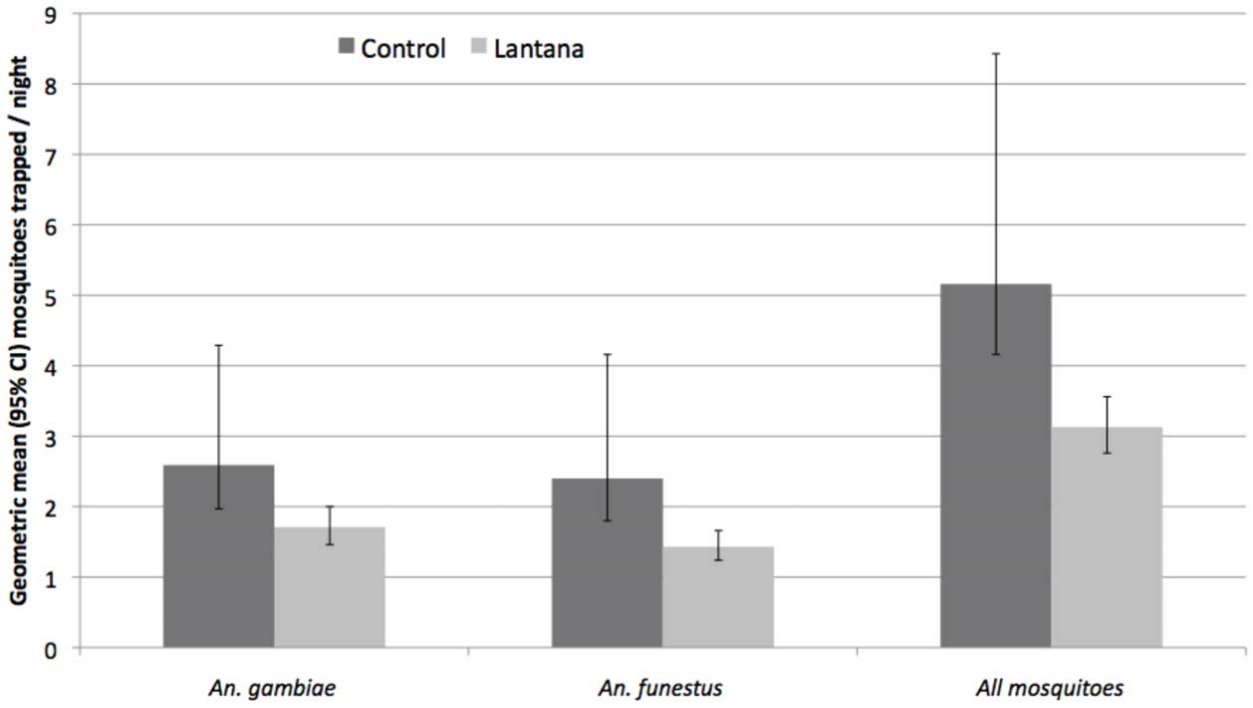
Geometric mean and 95% Confidence Intervals (95% CI) mosquitoes collected per night using CDC LT between September 2008 and July 2009 in houses with *Lantana camara* (Lantana) planted around them and houses without repellent plants (Control).

Multivariable analysis, demonstrated the protective effect of Lantana relative to control with reductions in indoor density of *An. gambiae* s.s. by 56% (IRR 0.44, 95% CI 0.28–0.68, p<0.0001); *An. funestus* s.s. by 83% (IRR 0.17, 95% CI 0.09–0.32, p<0.0001), and total mosquitoes by 50% (IRR 0.50, 95% CI 0.38–0.67, p<0.0001) relative to those houses with no plants (Figure 4 and Table 3). Most houses with Lantana (80%) had greater than 25% coverage of plants that were between 40 cm and 1.5 m high around the walls of the house. It is likely that as the plants grow their protection will increase. However, this could not be evaluated because the study was stopped when indoor residual spraying was introduced into the village as part of the National Malaria Control Program in September 2009. The study had to be discontinued because IRS would severely confound any inference about the effect of the plants on indoor mosquito density.

### Perception of mosquitoes and strategies to avoid mosquito bites

During the baseline data collection participants were asked to describe ways in which malaria may be prevented. Most respondents mentioned some means of mosquito related prevention with 41% mentioning bednets, 14% mentioning filling in puddles and cleaning the environment, 4% mentioned repellent plants, 1% mentioned spraying insecticides. However, 12% of participants thought that you can prevent malaria by being cleaner or eating clean food and 21% did not know how it is prevented. When asked what causes malaria, 81% identified mosquitoes. There was a clear link in the mind of the participants between mosquitoes and an untidy environment, and 60% of participants knew that mosquitoes breed in puddles and standing water.

When asked how they stop themselves being bitten by mosquitoes, 79% said that they covered themselves with long clothes, 52% said that they used bednets, 14% mentioned house spraying with insecticide 6% sat close to a smoking fire and 2% burned repellent plants. Many people modified the environment around their homes by filling in puddles (65%) and clearing vegetation (79%). Over half of households did not have a bednet of any kind (53%) and only 29% of the households interviewed had sufficient bednets to cover all children under five living in that house with 9% of households having sufficient bednets for all household members. The difference in the number of household heads who reported using bednets (53%) versus the number of households that actually owned bednets (47%) might reflect people identifying a means of protection versus one that they actually were able to use.

### Qualitative research with community participants

The qualitative research found a broadly favourable response to the Lantana plants, with some reservations, and many people asked for more plants to be planted. There were perceived benefits of the plants in reducing the number of mosquitoes in their houses, being used as ornamental plants and for herbal medicines, as illustrated in the following quotation:

*The plants are used as medicines to soothe chest problems when added into water or tea…. As a result of these plants mosquitoes and malaria have decreased. (Ambrose, adult man focus group discussion)*

This is in contrast to the generally negative opinion about indoor residual spraying where many people commented on how it was felt to lead to more fleas inside the houses, causing disturbance to householders during the night. A typical comment was:

*Fleas, fleas, fleas…….fleas …brings fleas, oh my God! We were not able to sleep at night after spraying houses (Chausiku, adult woman, focus group discussion)*

However there were also a number of negative views associated with the planting of Lantana that had an impact on villagers' livelihoods. First, the plant is considered a weed, grows very fast and is invasive of fields and cleared areas. Controlling the growth of the plant has implications for people's workloads where family labour is in short supply, and adds to the burden of weeding when it invades cultivated crops. Another common complaint was that the plants, have an impact on the yields of their crops, as explained in this quotation:

*These plants spread so fast in our farms and compete with our banana crops for fertility. The plants cause banana trees to become thin and tall, and as result banana crops produce poor yield (Kaketi, young man, in-depth interview)*

A second issue was uncertainty about what is causing the reduction in mosquitoes. The qualitative research was conducted at the end of the evaluation. Thus there have been several interventions at the same time including residual spraying started in July 2009 and insecticide treated bednets and thus it is impossible to assess how much a reduction in malaria transmission is related to the planting of Lantana. It is impossible to attribute this perceived effect to the Lantana since the research did not monitor mosquito densities in sentinel houses before and after introducing Lantana into the community. The results from the study revealed that there were perceived feelings of the decrease of mosquitoes inside houses, and lowering of the incidence of malaria. Other reasons given for the perceived reduction in mosquito numbers and malaria included an expansion of settlement where bush was cleared for new homes and farms, the IRS programme and the repellent plants. This ambivalence is illustrated in the following quotation:

*Nowadays mosquito nuisance has decreased so much. However, we are not certain whether it is the plants or sprays [IRS] which has caused this situation (Grace, adult woman, focus group discussion)*

It was also revealed that the rejection of the IRS programme by some people could be partly attributed to using repellent plants as an alternative. As expressed in one of the focus groups:

*“the presence of repellent plants encourage many people to reject the indoor residual spray program” (Bakari, adult man, focus group discussion).*

In addition, villagers were confused about who was implementing the IRS programme, and many thought it was the NGO Concern and not the government.

A final point to consider in understanding responses from the villagers is that they are aware that Concern has been responsible for providing piped water to the community, which people appreciate and may not want to jeopardise by criticising an additional activity. Some of the researchers were also employed by Concern, and thus not able to be neutral in the eyes of the local population. Thus, people would be constrained from being too critical of what they see as a useful organization. Given these issues a second period of qualitative research with the villagers will be conducted, including observation of the growth and maintenance necessary for Lantana plants, and further in-depth discussions and interviews regarding the impact on livelihoods and labour.

## Discussion

This is the first community study using live plants under user conditions as a physical and chemical barrier to prevent mosquitoes entering rural homes. The results are extremely encouraging with a strongly statistically significant reduction in both of the primary malaria vector mosquitoes *An. gambiae* s.s. (56%) and *An. funestus* s.s. (83%); in addition to nuisance mosquitoes, which is important to ensure user acceptability through a perceived reduction in mosquito bites [16]. Data were collected under real-life conditions, with a large number of potential sources of variation, but Lantana was consistently associated with lower indoor densities of mosquitoes. Households were assigned treatments randomly and only sampled once to prevent the bednets distributed to those houses from which we sampled mosquitoes confounding the results. This means that data were more variable than if we had conducted a repeated measures design, however the Lantana still had a consistent effect. The strong association between mosquitoes as a cause of malaria by community members, but low use of bednets and high use of environmental management indicates that people want to reduce mosquitoes but are unable to afford the means to do so. For this reason, the use of plants may be useful in this region.

Lantana has several important qualities that make it effective in preventing mosquito house entry. It contains a variety of terpines and alkaloids, including high quantities of caryophylene [18] that has good repellent efficacy against *An. gambiae* s.s. [19]. In addition to caryophyllene, the essential oil derived from the leaves contains high quantities of eucalyptol, alpha-humelene and germacrene that are toxic to mosquito adults [20]. The efficacy of Lantana as a mosquito repellent has been demonstrated by a number of authors, the most notable of which was a controlled field study conducted in Kenya with the same vector species [21]. In this study, Seyoum *et al* used ten potted plants hung close to the eaves of four houses over 24 nights, and also used CDC LT as a proxy for human exposure to host seeking mosquitoes [22]. The authors demonstrated a 27.22% (95% C.I. 0.04–47.16) reduction in house entry of *An. gambiae* s.l. (mainly *An. arabiensis*) which is half that observed in our study. The Kenyan study [21], did not demonstrate repellent efficacy against *An. funestus*, contrary to the significant 83% reduction seen in this study. The reason for this difference may be related to mosquito density as the average nightly catch of anophelines in the Kenyan study was >300, whereas we collected <5 anophelines per night. In addition, Seyoum *et al.* noted that *An. funestus* in Western Kenya have low sensitivity to repellents [23]. However, it is important to consider that the plants used in this study were over 80 cm tall and as such will have emitted a greater amount of volatile compounds than those potted plants used by Seyoum.

Lantana has several unusual features that contribute to its repellency. Lantana emits very large amounts of volatile organic compounds from the leaves [24], [25] including α-pinene that is a known mosquito repellent [26]. The α-pinene emission from live Lantana is almost an order of magnitude greater than that emitted from live Eucalyptus and warrants further study as it may explain the ability of undamaged Lantana to repel mosquitoes (as opposed to most plants that require some mechanical damage to promote release of repellent “green volatiles” [27]).

Of additional importance, there is a well-researched body of evidence indicating that mosquitoes feeding on Lantana flowers have reduced survival [28], [29], [30]. This is important because increasing mosquito access to sugar could enhance their survival. Both male and female mosquitoes feed on sugar as a source of energy but the role of plant feeding in the biology of *An. gambiae* is poorly understood [31]. Sugar is used by both sexes for energy and sugar meals may play an important role in enhancing the ability of mosquitoes to transmit malaria parasites by extending female lifespan [32]. This was recently demonstrated from a field experiment where populations of *An. sergentii* with better access to sugar resources were more likely to transmit malaria. The authors demonstrated that mosquito survival was enhanced so that a greater proportion of mosquitoes survive long enough for the malaria parasite to develop, and the period between blood feeding events was shortened increasing the probability that they are infected or infect a human when blood-feeding [33].

Indeed, under natural conditions where breeding sites are a limiting resource, teneral *An. gambiae* may require multiple blood meals to develop eggs [34], especially those mosquitoes that are smaller due to larval development under sub-optimal conditions [35]. *An. gambiae* is opportunistic in its use of blood and sugar for a first meal, depending on the availability of either resource [36]. When sugar and blood are both available, females exhibit a preference towards taking sugar meals in the period immediately following emergence [37], indicating that sugar feeding must improve mosquito fitness. *An. gambiae* is able to use sugar or blood to enhance lipid reserves that may be used for oogenesis [38], to provide energy for flight [39] and mating [40]. This explains why meal preference is opportunistic; although the preference for sugar after eclosion when both are available may be because obtaining a sugar meal carries a lower risk than from some vertebrate hosts [41] and may reduce foraging related mortality [42]. The availability of sugar has important epidemiological ramifications because the absence of sugar increases the number and frequency of blood feeds that are taken from man [43], increasing vectoral capacity [44].

Lantana may reduce vectoral capacity in two ways. First, its repellent properties reduce man-vector contact, especially in a setting, such as in Kagera, where the primary vectors *An. gambiae* s.s. and *An. funestus* s.s. feed indoors and therefore reduce mosquito access to blood. Second, Lantana has been shown to be discriminated against by sugar seeking mosquitoes, although they will feed on it in the absence of alternatives [45]. However, the probability of mosquitoes sugar feeding on Lantana under field conditions has not yet been measured. Those mosquitoes that feed on Lantana in the laboratory have lower survival [29] and lay fewer eggs [28] than mosquitoes that feed on other sugar sources including domestic plants [45]. Therefore, the introduction of Lantana will not improve the survival of mosquitoes, or consequently increase their ability to transmit disease. This negative effect on mosquito survival is a highly desirable characteristic for any vector control tool as it reduces the population size of the vector and the probability that mosquitoes will live long enough to transmit the malaria parasite. Therefore, even those not using the plant to prevent mosquitoes entering their homes may benefit from the “community effect” on malaria transmission. However, a large community based study to measure the influence of Lantana on the mosquitoes and clinical outcomes would be necessary to measure such a potential effect.

The most important quality of repellent plants as a concept for household protection is that they are extremely cheap, widely available and they are self-sustaining. Lantana originates from South America but was introduced as an ornamental garden plant into Africa in the mid-19^th^ Century [46] and is now naturalised in many African countries including Kenya, Uganda and Tanzania. As Lantana grows wild in Tanzania, it can be provided to a household averaging 5 people for an initial outlay of US $1.50, covering the costs for plants to be grown in the nursery, transported and planted around houses, making it an extremely economically attractive compliment to existing malaria control strategies. Lantana is extremely tolerant of drought and frost, survives up to 2000 m above sea level and grows well without being tended. Maintenance required once the plants are established is to prune back the plants when they become too large. Therefore, the duration of protection is continuous after the plants reach sufficient height to impede mosquito entry into homes via eaves, window and cracks in walls. Thus, with minimal compliance, a household is provided with a means of preventing mosquito house entry that protects throughout the year on a continuous basis with resources available in the community.

For better community acceptance, it is advantageous if the repellent plant chosen has multiple benefits. Lantana is pleasing to look at and for this reason is a common garden ornamental. It is also useful as a hedge as it is dense with prickles, and planting it close to the windows can improve the security of the home. Lantana may be used as a leaf mulch to prepare the ground for crops and it improves the fertility of rocky, gravel, or hard lateritic soils, enriches the soil as the ash is rich in potassium and manganese, serves to retain humus in deforested areas and checks soil erosion [18]. Lantana twigs and stems serve as useful fuel for cooking and the use of Lantana as firewood serves to reduce the burden on native forests, especially in Ngara where the influx of over half a million refugees in the 1990s caused severe deforestation and environmental degradation [47].

However, Lantana is not native to East Africa and has caused significant problems in several areas where it invades native or agricultural ecosystems [48]. Lantana is now a major weed in many regions of the tropical areas of Africa and Asia, in particular island ecosystems. The plants can grow individually in clumps or as dense thickets, crowding out more desirable species. Fruit-eating birds are the means by which the seeds of Lantana are dispersed. This is an efficient means of dispersal, allowing the rapid dissemination of the plants throughout a wide area, aiding the plant's invasive potential over long distance. Its use cannot be advocated in areas where it might become invasive and so we advocate that its use be absolutely restricted to those areas where it has been localised without harm to local vegetation. Importantly, several sterile ornamental varieties have been developed, including those that do not produce berries and these should be further investigated for mosquito prevention. Cattle do not readily eat Lantana unless pasturage is very scarce, but if they do feed on it then the plant is toxic [49]. In tropical countries, the ripe blue-black berries are eaten, and the green berry causes stomach upset though the fruit is not dangerous to humans [50]. In addition to its propensity for dominating ecosystems, Lantana has been recorded as a refuge for Tsetse flies [51] and must therefore not be widely planted without a thorough ecological assessment. Lantana at the field site did occasionally die as a result of attack from pests. Spread was reduced by farmers who burn their fields before planting crops, as is standard practice in Tanzania. However, in the study village, Lantana invaded a banana plantation, and had to be removed by local farmers.

The rapid year-round growth and climatic tolerance that makes it invasive is precisely the characteristics that make it a useful repellent plant. It does not die back. However, as discussed in the previous section these attributes could have a serious impact on the livelihoods of subsistence farmers with little margin for additional labour. Food shortage is common and family labour is not sufficient for current demands. The two issues to address are the invasive tendency of the plant and the amount of labour needed for weeding/pruning, and issues around appropriate tools for pruning the plant regularly. In an area where some children continue to provide family labour, it is imperative that additional workload issues are studied. The qualitative research uncovered how many people experience hard continuous field labour that reaps insufficient nutritious food and income for an adequate standard of living that thus has a direct impact on health and wellbeing. Attitudes to health and ill health were fatalistic. While having malaria was seen as an inevitable part of life to villagers– in contrast to the strong risk adverse attitude of the Western mind – additional labour would be viewed as a serious and harmful change in their wellbeing.

Lantana as a complement to existing malaria control strategies shows excellent potential, provided an ecological assessment can demonstrate that it will not become invasive or proof that a sterile variety can prevent mosquito house entry. The plant has interesting and unusual modes of action in the barrier effect it provides through dense foliage and the emission of large amounts of volatile terpines. Other unique properties are the deleterious effect that the sugar of Lantana has on mosquito survival and reproductive fitness. Although plants do not provide the level of protection that one would expect from house screening being an imperfect system, they do provide a medium level of protection. This method has several desirable features: 1) maximal unit efficacy providing household protection for a small initial outlay, 2) long-lasting protection, 3) general community acceptance and uptake, and 4) low individual requirements for compliance outside of maintaining the plants. People in developing countries often use plants to repel mosquitoes simply because they are cheap and available [52], [53] and it should be remembered that a method which is freely available but of small benefit may be more useful than one which is more effective but unaffordable.

## Materials and Methods

### Ethics Statement

Ifakara Health Institute Institutional Review Board IHRDC/IRB/No. A003 and the Tanzanian National Institute of Medical Research NIMR/HQ/Vol.IX/654 granted ethics approval for the mosquito collection and interviews. All information from questionnaires was collected upon written informed consent using an identification number to preserve participant confidentiality. All participants benefitted from participation through the receipt of an insecticide treated bednet. The qualitative research was granted ethical approval by the local academic ethics committee at Durham University.

### Study area

Research was performed in a village (2° 33′S, 30° 49′E) in Ngara District, part of the Kagera Region in northwest Tanzania. The village is located at 1,500 m altitude with the population comprising two main tribes Wahangaza and Washubi who engage predominantly in small-scale farming and to a lesser extent livestock keeping. The main food crops grown include: bananas, beans, cassava, potatoes, yams and maize. Cash crops are coffee, cotton and tobacco. Although the district has some rain throughout the year, there are two rainy seasons with most of the rain falling between the months October to November and March to May, with annual rainfall around 1,400 mm; rainfall and temperature strongly influence monthly malaria incidence [54].

### Community sensitisation

A village was selected as it is close to Concern's Ngara Office and was recruited upon community sensitisation and permission from village leaders. During the initial preparation period, meetings were held with the village leaders including District Medical Officer, mwenyekiti (Village head) and mabalozi (village representatives). Once permission to conduct the study was obtained from the village leaders in the selected village, a community sensitisation workshop was held in June 2008 with approximately 60 participants. The workshop introduced the study objectives and methodology including demonstrations of mosquito collection procedures and answered questions that the community had about the study. A second community motivation meeting was held in May 2009 to remind participants of the importance of the study and to answer any concerns or questions that they have.

### Sampling design

Concern assisted those families within the villages who requested Lantana establish the plants around their homes, especially to cover the windows and the eaves of the houses, which are known mosquito entry points. As plants were allocated only to those families that requested them, they were therefore allocated at random. Houses were selected in a stepwise fashion starting from a random start-point and moving to the nearest house that consented to participate. Within the study period data was collected from 231 houses with, and 90 houses without repellent plants.

### Selection of Repellent plants

Those plants were grown in the Concern Repellent Plant Nursery (Figure 1), developed as part of the “Provision of Water, Sanitation, Vector control & Hygiene promotion for refugees in Lukole A & B”. After UNHCR (United Nations High Commission for Refugees) stopped Indoor Residual Spraying in the Lukole refugee camp in Ngara District in 2003 due to budget constraints, malaria prevalence increased. The plants were traditionally used to repel mosquitoes by the Burundian Refugees in the camp and were introduced by Concern in an attempt to address the increasing malaria problem. Some Concern staff had past experience in using repellent plants in their home villages identified those plants used by the refugees to repel mosquitoes and established them in a nursery. After anecdotal reports of excellent plant efficacy against mosquitoes from the camp Concern initiated the pilot efficacy study.

### Questionnaires

Questionnaires were designed to capture information on house design, socioeconomic status, malaria prevention knowledge, attitude and practices. These were developed in English with translation into Kiswahili and back-translation into English to ensure appropriate meaning. The questionnaires were field-tested with the local population to check for ambiguity before the final version was used for the study. Questionnaires were administered to household-heads over 18 years upon written informed consent.

### Mosquito collection

Mosquito collection was performed using Centers for Disease Control light traps (model 512 John Hock) (CDC LT), which was hung by the feet of an individual [55] sleeping under an untreated bednet (Safi Net) that was provided by the study. Untreated bednets were used to avoid confounding the study due to the repellent effect of insecticide treated bednets [56]. However, on the morning when the trap was collected, the householders were assisted in treating their bednet with insecticide (KO tab (Bayer), local name Ngao) by Concern staff and the bednet was given to that household to ensure that participation in the study was beneficial to participants. Collections were performed between 19.00 hrs and 07.00 hrs. Mosquitoes collected in the traps were taken back to the Concern laboratory where they were killed using ethyl acetate and sorted. Anopheles mosquitoes were separated, placed in eppendorf tubes with silica, labelled and sent to IHI for further speciation using polymerase chain reaction (PCR) [57], [58]. Collection frequency varied due to logistical issues, but averaged eight trap nights per week.

### Data analysis

Data were double entered into a laptop using an Epi Info™ version 3.5 (Centres for Disease Control) template that corresponded to the format of the questionnaires. The template had drop down lists of legal values that allows for automatic coding of the data ready for analysis and reduces sources of human error with its user-friendly interface. Data were cleaned using STATA 11 IC (StataCorp) to check for lack/excess of data, inconsistencies and outliers [59].

Data on frequency of factors that were assumed to influence indoor mosquito density were described and tested for heterogeneity between the two treatment arms (Lantana and control) using a two tailed Pearsons Chi-Squared test. Mosquito count data were over dispersed with the variance more than two times greater than the mean. Therefore, data were analysed with univariable generalised negative binomial regression with robust standard errors. Test of the model with Likelihood Ratio test showed that the assumption of deviation from a Poisson distribution was appropriate. Variables that were assumed to influence indoor mosquito density were included as factors with the exception of numbers of people in the house, numbers of bednets owned and numbers of bednets used that which were included as continuous variables. A backwards-stepwise model building procedure was used: those variables that were statistically significant or approaching significance (P<0.1) in the univariable analysis were included in the multivariable analysis. Those variables in the multivariable analysis that did not approach significance at p = 0.05 were removed until only significant variables remained in the final analysis. Risk factors for mosquito house entry are presented as Incidence Rate Ratios (IRR) with 95% confidence intervals (CI).

### Qualitative social science research with villagers

In addition to the quantitative survey, qualitative methods were conducted in two villages in the study area in order to assess the impact of the various interventions and the attitudes and perceptions of the villagers. One of us (JJS) lived in the village for four months in 2010, conducting 15 focus groups with men, women and village leaders and 5 semi-structured interviews, as well as conducting general observation of village life and many informal discussions. Discussions with participants covered a range of issues, including health and wellbeing, livelihoods and social issues, including views of a range of interventions, such as repellent plants. Respondents were selected from those individuals who had been involved in either the repellent plant project and/or the indoor residual spray programme. The respondents were recruited through *mabalozi* (village representatives) in collaboration with each *mwenyekiti wa kitongoji* (sub-village chairman). All research participants were provided with verbal explanations of the study, which emphasized confidentiality, anonymity, and the option to withdraw from the study at any time. In return for their time, people were given refreshments and a small cash payment to compensate for their travel costs. The discussions were conducted in Swahili, with both Swahili and English transcriptions. With the few individuals who were not able to speak Swahili, *and if speaking in a local dialect*, an interpreter was used to facilitate the discussion. The transcripts were coded, maintaining the anonymity of the respondents, and analysed according to emerging themes and issues, and making note of the representativeness of responses. Empirical data was analysed finding patterns that inductively lead to a grounded theory approach. The villagers' views of the interventions (the repellent plants, IRS, and treated bednets) were embedded in a wider analysis of their perceptions and actions in regard to their health and wellbeing, environmental risks to health, including malaria, and the wider vulnerabilities of endemic poverty.

## Supporting Information

Table S1 Features of plants used by Burundian Refugees to repel mosquitoes.
(DOCX)
