# Supplementary material for: Repellent Plants Provide Affordable Natural Screening to Prevent Mosquito House Entry in Tropical Rural Settings—Results from a Pilot Efficacy Study
Source: PLoS One. 2011 Oct 12;6(10):e25927. doi: 10.1371/journal.pone.0025927 (PMC3192125; doi:10.1371/journal.pone.0025927)
Supplement: Table S1 — Features of plants used by Burundian Refugees to repel mosquitoes. (DOCX) [file pone.0025927.s001.docx]

SOM 1 Features of plants used by Burundian Refugees to repel mosquitoes

| Botanical Name | Local Name | | Description | Local Uses | Repellent compounds | Tested mode of use | % Repellency | Type of test | Suitability |
| --- | --- | --- | --- | --- | --- | --- | --- | --- | --- |
|  | Language | Name |  |  |  |  |  |  |  |
| *Lantana camara* L.  Verbenaceae | Kihangaza  Kiswahili  English  Kirundi | Umuhengeli  Mutululu Mvuti  Lantana  Umuheng-erihengeri | Origin South America. Naturalised throughout East Africa  Erect vigorous shrub with prickles;  1.2-2.4 metres tall; strong root system;  0- 2000m altitude; tolerates variety of soils;  tolerant to frost and drought;  prefers open un-shaded situations e.g. wastelands, rainforest edges, beachfronts, forests recovering from fire or logging [1] | Cough [2]  Stomach ache  Syphilis Gonorrhoea [3]  Mosquito repellent [4] | αpinene, caryophyllene, cineole, citral, eugenol, geraniol, linalool, p-cymene, terpeneol, and up to 80% caryophyllene [5] | Potted plant | 32.4% protection *An. gambiae* s.s | Semi-field study in Kenya [6] | Repellent in intact, live format  Vigorous, hardy and grows all year round with minimum care.  Can provide alternate firewood and protect soil in deforested areas.  Some hybrids are serious invasive pests – ecological assessment is required |
|  |  |  |  |  |  | Potted plant | 27.22% protection *An. gambiae* s.l. | Field study in Kenya [7] |  |
|  |  |  |  |  |  | Flower extract in coconut oil | 94.5% protection *Ae. aegypti* and *Ae. albopictus* for one hour | Laboratory study [8] |  |
|  |  |  |  |  |  | Periodic thermal expulsion (leaves) | 42.4% protection *An. gambiae* s.s | Semi-field study in Kenya [9] |  |
|  |  |  |  |  |  | Mosquitoes allowed to sugar feed on plants | *An. gambiae* s.s survival reduced by 42% and fecundity reduced by 47% | Laboratory assay [10] |  |
| *Ocimum americanum* L.  Lamiaceae | Kihangaza  Kiswahili  English  Kirundi | Nabahengele, kivumbasi  Hairy Basil  Isonga | Found throughout tropics  Perennial herb  15-50 cm tall  Tolerates 7-36°C  Grows 500-2000m  Prefers sheltered conditions | Stomach ache and mosquito repellent [11]  Cough [12] | p-cymene estragosl  linalool linoleic acid  eucalyptol eugenol  camphor  citral thujone  limonene  ocimene  [13] | Potted plant | 39.70% protection against *An. gambiae* s.s | Semi-field study in Kenya [6] | Repellent in intact, live format  Dies back in dry season  Does not grow sufficiently high to protect eaves as mosquito entry point |
|  |  |  |  |  |  | Potted plant | 37.91% protection against *An. gambiae* s.l. | Field study in Kenya [7] |  |
|  |  |  |  |  |  | Fresh plants combined with O. suave bruised and applied topically | 50% protection against *An. gambiae* s.l. | Field study in Tanzania [14] |  |
|  |  |  |  |  |  | Periodic thermal expulsion (leaves and seeds) | 43% protection against *An gambiae* s.s | Semi-field study in Kenya [9] |  |
|  |  |  |  |  |  | Periodic direct burning (leaves and seeds) | 21% protection against *An. gambiae* s.s | Semi-field study in Kenya [9] |  |
|  |  |  |  |  |  | 100% essential oil combined with vanillin 5% applied topically | 100% protection against *Ae. aegypti* for 6.5 hours  100% protection against *Cx. quinquefasciatus* for 8 hours  100% protection against *An. dirus* for 8 hours | Laboratory study [15] |  |
| *Ocimum kilimandscharicum* Guerke  Lamiaceae | Kihangaza  Kiswahili  English  Kirundi  Kihaya | Akashwagara | Native to East Africa.  Evergreen aromatic perennial shrub  Grows up to 2 m high  Grows up to 900 m altitude  Tolerates rainfall up to 1250 mm  Strong root system | Eye infections [16]  Stomach upsets  Blocked nose [72]  Mosquito repellent [11] | Limonene  1,8-Cineole  Camphor  Linalool  4-Terpineol  Trans-caryophyllene  camphor  [16] | Thermal expulsion  (leaves and seeds) | 44.5% protection against *An. gambiae* s.l. | Field study in Kenya [7] | Frequently used as a boundary or hedge around Tanzanian homes.  Prevents soil erosion where it is grown  Easy to grow  Not repellent in intact, live format |
|  |  |  |  |  |  | Thermal expulsion  (leaves and seeds) | 37.63% protection against *An. funestus* | Field study in Kenya [7] |  |
|  |  |  |  |  |  | Periodic thermal expulsion (leaves and seeds) | 52.0% protection against *An. gambiae* s.s. | Semi-field study in Kenya [9] |  |
|  |  |  |  |  |  | Potted plant | Not repellent | Semi-field study in Kenya [6] |  |
| *Ocimum suave* Willd  Lamiaceae | Kihangaza  Kiswahili  English  Kirundi | Tree basil | Originates from Africa, naturalised throughout tropics  Aromatic, perennial herb,  1-3 m tall; stems erect;  grows at altitudes 0-1500 m.  Coastal scrub, along lake shores, in savannah vegetation, in submontane forest, and disturbed land. |  |  | Thermal expulsion  (leaves) | 73.6 % protection from *An. arabiensis*  75.1% protection from *An. pharaoensis* | Field study in Ethiopia [18] | Used to mark boundaries or cultivated as a hedge.  Not repellent in intact, live format. |
|  |  |  |  |  |  | Direct burning  (leaves) | 71.5 % protection from *An. arabiensis*  79.7% protection from *An. pharaoensis* | Field study in Ethiopia [18] |  |
|  |  |  |  |  |  | Periodic thermal expulsion (leaves and seeds) | 53.1% protection from *An. gambiae* s.s. | Semi-field study in Kenya [9] |  |
|  |  |  |  |  |  | Potted plant | Not repellent | Semi-field study in Kenya [6] |  |
| *Cymbopogon citratus* L.  Poaceae | Kihangaza  Kiswahili  English  Kirundi | Chai Chai  Lemon grass | Originates from India but naturalised throughout the tropics  Clump forming grass; up to 1m tall  Grows up to 1400m altitude  Tolerant of a wide range of climates  Drought tolerant  Can tolerate shallow and poor soil | Inhaled to help influenza [19]  Medicinal tea for fever [20]  Mosquito repellent applied to skin [21] | citral, geranial, neral and beta-myrcene [22] | topically | 74% protection against *An. darlingi* for 2.5h  95% protection against *Mansonia* spp. for 2.5 hours | Field study in Bolivia [23] | Can be used to reduce erosion  Simple to propagate by division  Does not grow sufficiently high to protect eaves as mosquito entry point |
|  |  |  |  |  |  | Methanol leaf extract applied topically (2.5mg/m2) | 78.8 % protection against *An. arabiensis* for 12 hours | Laboratory evaluation [24] |  |
|  |  |  |  |  |  | 100% essential oil combined with vanillin 5% applied topically | 100% protection against *Ae. aegypti* for 6.5 hours  100% protection against *Cx. quinquefasciatus* for 8 hours  100% protection against *An. dirus* for 8 hours | Laboratory evaluation [15] |  |
| *Azadirachta indica* A. Juss  Meliaceae | Kihangaza  Kiswahili  English  Kirundi | Mwarobaini  Neem Tree | Originated in Myanmar and now naturalized throughout the tropics  small to medium-sized tree, usually evergreen,  15 – 20m tall  0-1500 m altutude  Up to 40 deg. C,  Mean annual rainfall: 400-1200 mm  Drought and frost resistant  Reduces soil erosion  Tolerates poor soil  Grows in most habitats |  | Azadirachtin | Direct burning  (leaves) | 76% protection from mosquitoes for 2 hours | Field study in Guinea Bissau [25] | Easy to grow and tolerant of wide variety of climates and soils  Provides wood and binds soil in deforested areas  Good source of wood and leaves / fruits used for many uses  Repellent constituents are not very volatile – need heat to volatilize them – not repellent in intact, live format |
|  |  |  |  |  |  | Periodic thermal expulsion (leaves) | 24.5% protection from *An. gambiae* s.s | Semi-field study in Kenya [9] |  |
|  |  |  |  |  |  | 1% neem oil volatilized in a kerosene lamp | 94.2% protection from Anopheles spp.  80% protection from Culex spp. | field study in India [26] |  |
|  |  |  |  |  |  | 2% neem oil applied topically | 56.75% protection from mosquitoes for 4 hours | field study in Bolivia [27] |  |

1. Day M, Wiley CJ, Playford J, Zalucki MP (2003) Lantana: current management status and future prospects ACIAR Monograph 102. Australian Centre for International Agricultural Research.

2. Polygenis - Bigendako MJ (1990) Recherches ethnopharmacognosiques sur les plantes utilisées en médecine traditionnelle au Burundi occidental. Thèse présentée en vue de l'obtention du grade de Docteur en sciences, année acad. 1989 - 1990, , Faculté des Sciences, Laboratoire de Botanique Systématique et de Phytosociologie, 352 p.

3. Moshi MJ, Otieno DF, Mbabazi PK, Weisheit A (2010) Ethnomedicine of the Kagera Region, north western Tanzania. Part 2: The medicinal plants used in Katoro Ward, Bukoba District. J Ethnobiol Ethnomed 6: 19.

4. Kweka EJ, Mosha F, Lowassa A, Mahande AM, Kitau J, et al. (2008) Ethnobotanical study of some of mosquito repellent plants in north-eastern Tanzania. Malar J 7: 152.

5. Ghisalberti EL (2000) *Lantana camara* L. (Verbenaceae). Fitoterapia 71: 467-486.

6. Seyoum A, Kabiru EW, Lwande W, Killeen GF, Hassanali A, et al. (2002) Repellency of live potted plants against *Anopheles gambiae* from human baits in semi-field experimental huts. Am J Trop Med Hyg 67: 191-195.

7. Seyoum A, Killeen GF, Kabiru EW, Knols BG, Hassanali A (2003) Field efficacy of thermally expelled or live potted repellent plants against African malaria vectors in western Kenya. Trop Med Int Health 8: 1005-1011.

8. Dua VK, Gupta NC, Pandey AC, Sharma VP (1996) Repellency of *Lantana camara* (Verbenaceae) flowers against Aedes mosquitoes. J Am Mosq Control Assoc 12: 406-408.

9. Seyoum A, Palsson K, Kung'a S, Kabiru EW, Lwande W, et al. (2002) Traditional use of mosquito-repellent plants in western Kenya and their evaluation in semi-field experimental huts against *Anopheles gambiae*: ethnobotanical studies and application by thermal expulsion and direct burning. Trans R Soc Trop Med Hyg 96: 225-231.

10. Manda H, Gouagna LC, Foster WA, Jackson JR, Beier JC, et al. (2007) Effect of discriminative plant-sugar feeding on the survival and fecundity of *Anopheles gambiae* Mal J 6: 113.

11. Weiss EA (1979) Some indigenous plants used domestically by East african costal fishermen. Econ Bot 33: 35-51.

12. Desouter S (1991) Pharmacopée humaine et vétérinaire du Rwanda.Musée royal de l'Afrique centrale Tervuren. Ann Soc Eco 22: 254.

13. Vieira RF, Grayer RJ, Paton AJ (2003) Chemical profiling of *Ocimum americanum* using external flavonoids. Phytochemistry 63: 555-567.

14. White GB (1973) The insect repellent value of *Ocimum* spp. (Labiatae): traditional anti-mosquito plants. East African Med J 50: 248-252.

15. Tawatsin A, Wratten SD, Scott RR, Thavara U, Techadamrongsin Y (2001) Repellency of volatile oils from plants against three mosquito vectors. J Vector Ecol 26: 76-82.

16. Ntezurubanza L, Scheffer JJ, Looman A, Baerheim Svendsen A (1984) Composition of essential oil of *Ocimum kilimandscharicum* grown in Rwanda. Planta Med 50: 385-388.

17. Kokwaro JO (1993) Medicinal plants of East Africa, 2nd ed. Nairobi: Kenya Literature Bureau.

18. Dugassa S, Medhin G, Balkew M, Seyoum A, Gebre-Michael T (2009) Field investigation on the repellent activity of some aromatic plants by traditional means against *Anopheles arabiensis* and *An. pharoensis* (Diptera: Culicidae) around Koka, central Ethiopia. Acta Trop 112: 38-42.

19. Tabuti JRS, K.A. Lye, S.S. Dhillion (2003) Traditional herbal drugs of Bulamogi, Uganda: plants, use and administration. J Ethnopharmacol 88: 19-44.

20. Namukobe J, Kasenene JM, Kiremire BT, Byamukama R, Kamatenesi-Mugisha M, et al. (2011) Traditional plants used for medicinal purposes by local communities around the Northern sector of Kibale National Park, Uganda. J Ethnopharmacol 136: 236-245.

21. Ntonifor NN, Ngufor CA, Kimbi HK, Oben BO (2006) Traditional use of indigenous mosquito-repellents to protect humans against mosquitoes and other insect bites in a rural community of Cameroon. East Afr Med J 83: 553-558.

22. Leal WS, Uchida K (1998) Application of GC-EAD to the determination of mosquito repellents derived from a plant *Cymbopogon citratus*. J Asia-Pacific Entomol 1: 217-221.

23. Moore SJ, Hill N, Ruiz C, Cameron MM (2007) Field Evaluation of Traditionally Used Plant-Based Insect Repellents and Fumigants Against the Malaria Vector *Anopheles darlingi* in Riberalta, Bolivian Amazon. J Med Entomol 44: *624-630*.

24. Karunamoorthi K, Ilango K, Murugan K (2010) Laboratory evaluation of traditionally used plant-based insect repellent against the malaria vector *Anopheles arabiensis* Patton (Diptera: Culicidae). Parasitol Res 106: 1217-1223.

25. Palsson K, Jaenson TG (1999) Plant products used as mosquito repellents in Guinea Bissau, West Africa. Acta Trop 72: 39-52.

26. Sharma VP, Ansari MA (1994) Personal protection from mosquitoes (Diptera: Culicidae) by burning neem oil in kerosene. J Med Entomol 31: 505-507.

27. Moore SJ, Lenglet A, Hill N (2002) Field evaluation of three plant-based insect repellents against malaria vectors in Vaca Diez Province, the Bolivian Amazon. J Am Mosq Control Assoc 18: 107-110.
